# Tumor Angiogenesis and Anti-Angiogenic Strategies for Cancer Treatment

**DOI:** 10.3390/jcm9010084

**Published:** 2019-12-29

**Authors:** Raluca Ioana Teleanu, Cristina Chircov, Alexandru Mihai Grumezescu, Daniel Mihai Teleanu

**Affiliations:** 1 “Victor Gomoiu” Clinical Children’s Hospital, “Carol Davila” University of Medicine and Pharmacy, 050474 Bucharest, Romania; raluca.teleanu@umfcd.ro; 2 Faculty of Engineering in Foreign Languages, 060042 Bucharest, Romania; cristina.chircov@yahoo.com; 3 Department of Science and Engineering of Oxide Materials and Nanomaterials, Faculty of Applied Chemistry and Materials Science, Politehnica University of Bucharest, 011061 Bucharest, Romania; 4 Emergency University Hospital, “Carol Davila” University of Medicine and Pharmacy, 050474 Bucharest, Romania; daniel.teleanu@umfcd.ro

**Keywords:** tumor, angiogenesis, cancer, blood vessels, anti-angiogenesis strategies, nanotechnology, chemotherapy, immunotherapy, nanomaterials

## Abstract

Angiogenesis is the process through which novel blood vessels are formed from pre-existing ones and it is involved in both physiological and pathological processes of the body. Furthermore, tumor angiogenesis is a crucial factor associated with tumor growth, progression, and metastasis. In this manner, there has been a great interest in the development of anti-angiogenesis strategies that could inhibit tumor vascularization. Conventional approaches comprise the administration of anti-angiogenic drugs that target and block the activity of proangiogenic factors. However, as their efficacy is still a matter of debate, novel strategies have been focusing on combining anti-angiogenic agents with chemotherapy or immunotherapy. Moreover, nanotechnology has also been investigated for the potential of nanomaterials to target and release anti-angiogenic drugs at specific sites. The aim of this paper is to review the mechanisms involved in angiogenesis and tumor vascularization and provide an overview of the recent trends in anti-angiogenic strategies for cancer therapy.

## 1. Introduction

Through the process of blood circulation, the cardiovascular system ensures the proper functioning of the body by accomplishing three major roles, i.e., nutrients, gases, metabolites, chemical mediators, and waste products transport to or from the cells, immune system and homeostasis maintenance, and body temperature and pH adjustment [1,2].

Anatomically, the vascular system can be divided into macro- and microcirculation. Subsequently, three different segments can be identified within the macrocirculation: the arterial segment, containing elastic and muscular arteries, the venous segment, and the lymphatic segment, which includes lymphatic vessels and capillaries. The microcirculation represents the main exchange area between the circulating blood and the peripheral tissues, through the networks of arterioles, capillaries, and venules interposed between the arterial and venous segments, which vary depending on the tissue type [3,4,5]. Specifically, the capillary networks are fed by the terminal components of the arterial system, the arterioles, and drained by the first ramifications of the venous system, the venules [5]. Additionally, this dynamic and complex system comprising up to 10 billion capillary beds also includes the surrounding interstitial fluid, the lymphatic channels, and the collecting ducts [4].

The cardiovascular system is the first functional organ system that forms in the embryonic development. There are two main processes involved in the formation of blood vessels, namely vasculogenesis and angiogenesis [6,7]. Vasculogenesis is the process of de novo blood vessel formation through the differentiation of endothelial precursor cells or angioblasts from the mesoderm, and the subsequent formation of primitive and uniform vascular structures, termed as capillary plexuses, that will finally develop into hierarchically organized arteries, veins, and capillaries [7,8]. By contrast, angiogenesis represents the process of vessel formation from existing vessels [7,9]. Initially, angiogenesis leads to the development of capillaries through the angiogenic expansion of the primary capillary plexuses, followed by the growth of the vascular tree in coordination with the physiological expansion of the surrounding tissues. In this manner, unique heterotypic interactions are created, which will further induce vascular adaptations, leading to an array of molecular and physiological variations, termed as endothelial heterogeneity. Subsequently, the vascular structures further mature and their diameter and wall thickness increase through a process called arteriogenesis. Specifically, the mural cells proliferate and further acquire specialized characteristics, such as contractility [9,10].

Among these mechanisms, angiogenesis plays a fundamental role in various physiological and pathological conditions, including wound healing and bone repair and regeneration, by reestablishing the normal blood flow and consequently the efflux of gases, nutrients, and growth factors [7,11,12]. Additionally, by regulating the viability, proliferation, and differentiation of newly developed tissue structures, angiogenesis represents a key element in tissue engineering and regenerative medicine applications [11,13].

However, tumor cells develop an angiogenic phenotype through which the proangiogenic mechanisms overwhelm the downregulating processes. As a consequence, endothelial cells enter a rapid growth phase that further leads to the development of an oxygen and nutrients reach the tumor microenvironment that supports the growth of the tumor and the dissemination to distant sites [14,15]. In this manner, anti-angiogenesis-based therapies, which involve introducing agents to reduce blood vessel formation in malignant tumors or chronic diseases, have attracted great interest as potential anticancer treatments [11]. Therefore, the aim of this paper is to provide an overview of the mechanisms involved in physiological and pathological angiogenesis and the current state of anti-angiogenic therapies [7,12].

## 2. The Mechanisms of Angiogenesis

As previously mentioned, angiogenesis is the physiological process involved in the formation of blood vessels from pre-existing ones. Although it primarily occurs in embryonal and fetal development, it can also happen in adults, in the context of physiological adaptations, such as wound healing, muscle growth, organ lining regeneration, menstrual cycle through the growth of the endometrium, and placenta growth [16,17].

### 2.1. Angiogenesis Processes

Angiogenesis is the dynamic process through which new blood vessels are formed from pre-existing ones [16]. There are three types of angiogenesis, namely sprouting angiogenesis, which is the most common, intussusception or splitting angiogenesis, where a new wall grows inside an existing vessel, eventually dividing into two vessels, and looping angiogenesis, where vessel loops are mechanically dragged into the tissue [16,17].

The process of angiogenesis is strictly controlled during physiological processes, such as wound healing, tissue growth, and the female reproductive cycle. Therefore, in adult tissues, angiogenesis is mostly uncommon, and the endothelium is generally stable. In the resting vasculature, there are approximately 0.5% endothelial cells exhibiting mitotic activity and the angiogenic process requires the transition of endothelial cells from the quiescent state to an activated one [18].

The mechanism of angiogenesis involves several stages and a set of growth factors, substrate molecules, and multiple cell types [19]. The process is initiated and mediated by platelets, which facilitate a highly responsive system for regulating localized angiogenesis. By circulating through the body, platelets collect various angiogenesis-related proteins and store them in different compartments called alpha-granules. In the event of an injury, platelets are recruited to the wound site and become active, undergoing morphological changes and forming extensions, i.e., lamellipodia and filopodia, which facilitate clotting. In this manner, these clots block the injury and initiate the formation of new blood vessels through the sequential release of growth factors by degranulation [20,21]. Subsequently, to send signals for initiating, promoting, and terminating the formation of vessels, growth factors must bind glycosaminoglycans, such as heparan sulfate, onto the surface of the cells [22,23].

As the cells within the quiescent vessel sense the angiogenesis triggering signals, the surrounding pericytes detach from the vessel wall and the endothelial cells loosen their junctions, subsequently turning into motile tip cells, which are located at the growing ends of sprouting vessels and display long filopodia [19,24]. Sensing the proangiogenic directional cues in the environment, tip cells migrate in a specific direction [24]. Simultaneously, specific tip cells release inhibitory signals into the surroundings for preventing the uncontrolled migration towards the angiogenic signals. Tip cells are subsequently followed by stalk cells with fewer filopodia, which will extend, proliferate, and form the vascular lumen by producing adherent and tight junctions with adjacent endothelial cells for supporting the sprouting process [19,24]. Finally, for producing a functional and mature blood vessel, the migration and proliferation of endothelial cells are inhibited. Consequently, pericytes will form capillary walls and stabilize the new vascular tubes [18,19].

### 2.2. Angiogenesis Triggers

The initial step involved in angiogenesis involves the action of an angiogenic stimulus, such as hypoxia or inflammation [17]. Subsequently, a cascade of biochemical events are generated, and the repair of damaged vessels or the formation of the new vessel will be rigorously regulated by specific chemical signals, which are proteins known as angiogenic activators or inhibitors, that coordinate the functions of endothelial and smooth muscle cells [25,26].

The factors involved in angiogenesis can be categorized into environmental, mechanical, and chemical factors. Specifically, environmental factors include hypoxia or increased amounts of nitric oxide produced by endothelial cells, which will further stimulate the release of angiogenic triggers. Additionally, mechanical factors, namely hemodynamic and shear stress, have shown to stimulate the development of collateral vessel networks and maintain the patency of newly formed blood vessels [27]. Therefore, the mechanism of angiogenesis is mostly modulated by chemical stimuli, such as vascular endothelial growth factor (VEGF), fibroblast growth factor (FGF), platelet-derived growth factor (PDGF), angiopoietins (Ang), hepatocyte growth factor (HGF), hypoxia-inducible factor (HIF), insulin-like growth factor (IGF), transforming growth factor-beta (TGFβ), matrix metalloproteinase (MMP), and tumor necrosis factor (TNF) (Table 1) [19,22,27,28,29].

#### 2.2.1. Vascular Endothelial Growth Factor

VEGFs are a family of disulfide-linked soluble secretory glycoproteins found in higher eukaryotes. By binding to specific soluble, membrane-bound vascular endothelial growth factor receptors (VEGFR) or co-receptors, they modulate a series of responses, such as cell proliferation and migration, metabolic homeostasis, and tubulogenesis. Moreover, VEGFs rigorously control the physiological vasculogenesis and angiogenesis, the blood and lymphatic function in healthy or diseased adult tissues [30,31,32], and vascular permeability [33].

The VEGF family comprises a series of structurally and functionally related proteins, namely VEGF-A, VEGF-B, VEGF-C, VEGF-D, VEGF-E, and placental growth factor (PlGF) [30,32,34]. VEGF-A is the most studied member of the VEGF superfamily, as it is the key angiogenesis regulator and the most potent inducer of angiogenesis [31,34]. VEGF-A is selectively spliced to produce seven proangiogenic and five anti-angiogenic isoforms, e.g., VEGF-A_121_, VEGF-A_145_, VEGF-A_165_, VEGF-A_189_, and VEGF-A_206_ (indicating the number of amino acid residues in each polypeptide) [30,31,32]. VEGF-B is abundantly found in the cardiac and skeletal muscle, contributing to the pulmonary vascular remodeling after exposure to chronic hypoxia, VEGF-C and VEGF-D are key lymphangiogenesis regulators, and VEGF-E is an Orf virus-encoded VEGF homolog, which is not present in the human genome [34,35,36,37]. Most abundantly found in the placenta, thyroid, and lungs, the exact role of PlGF is unclear, with studies showing that it can act as an endogenous competitive inhibitor [34].

VEGFs exhibit their function by binding to three structurally related VEGFRs, namely VEGFR1 (FLT1), VEGFR2 (KDR, FLK1), and VEGFR3 (FLT4), which comprise three domains: an extracellular ligand-binding region with an Ig-like domain, a transmembrane domain, and a tyrosine kinase domain within the intracellular domain. VEGF binding to VEGFRs promotes the tyrosine kinase enzyme activation in the intracellular receptor domain and subsequently leads to the phosphorylation of the tyrosine residues, thus activating specific intracellular signaling pathways [30,32,38]. VEGFR1 is expressed in monocytes, macrophages, hematopoietic stem cells, vascular smooth cells, and leukemic cells, VEGFR2, in vascular endothelial cells, endothelial progenitor cells, and megakaryocytes, and VEGF3, in lymphoid endothelial cells [30,38,39]. VEGFs can also interact with other proteins, including neuropilins, integrins, cadherins, or heparan sulphate proteoglycans. Neuropilin co-receptors, specifically neuropilin-1 and neuropilin-2, are non-tyrosine kinase receptors and they selectively attach to VEGF isoforms, enhancing VEGFR1 and VEGFR2 functions by guiding the migration of endothelial cells in angiogenesis [38]. Maintaining physiologic levels of VEGFs in fundamental, as high concentrations have been related to aberrant angiogenesis and other pathological conditions [40].

#### 2.2.2. Fibroblast Growth Factor

FGFs are a family of 22 proteins, secreted by stem cells and damaged cardiac myocytes and vascular endothelial cells. FGFs have shown to induce endothelial cell differentiation, proliferation, migration, morphogenesis, and survival, extracellular matrix degradation by stimulating the secretion of proteases, such as plasminogen activator and metalloproteinases, and vessel maturation [41,42,43]. The angiogenic effects of the FGF family mostly rely on the activity of FGF-1 and FGF-2, which are key factors in wound healing, by stimulating the proliferation of endothelial cells and fibroblasts that will subsequently produce the granulation tissue necessary for the healing process [29,42,43].

FGF-1, also known as acidic FGF, is the broadest-acting protein of the FGF family and it can bind to seven fibroblast growth factor receptors (FGFR) subtypes. It is involved in the process of blood vessel formation as it stimulates the differentiation and proliferation of all the cell types necessary for creating the vessel wall. Although it is not as potent as FGF-1, FGF-2, also known as basic FGF, is a key pleiotropic regulator of vascular endothelial cell differentiation, proliferation, and migration and survival of blood vessel-specific cells. Inhibition of the FGF-2 signaling results in vascular endothelial cell junctions compromise and blood vessel increased permeability [43]. Besides angiogenesis, FGF-2 has a role in cardiac protection and regulates the differentiation of cardiac non-myocytes into functional cardiomyocytes [29].

The angiogenic activity of FGFs occurs through the binding to cell surface receptors, including tyrosine kinase FGFRs, integrins, and heparin sulphate proteoglycans [29], through the Ras/MAP-kinase pathway [42]. Moreover, the activity of FGF-2 is partially indirect, as the angiogenesis process is modulated by synergetic effects with various extracellular matrix-associated molecules, such as VEGFs and inflammatory cytokines and chemokines [29,41].

#### 2.2.3. Platelet-Derived Growth Factor

The PDGF family comprises four different polypeptide chains, which are inactive in their monomeric form, namely PDGF-A, PDGF-B, PDGF-C, and PDGF-D. After translation, PDGFs become active and exhibit their biological effects after dimerization, by binding of monomeric chains through amino acid disulfide bonds. There are four PDGF homodimers identified, namely PDGF-AA, PDGF-BB, PDGF-CC, and PDGF-DD, and one heterodimer, PDGF-AB [44,45]. Subsequently, the cellular effects are produced through the specific binding of the dimeric PDGF isoforms to two PDGF receptor (PDGFR) tyrosine kinases, PDGFR-α and PDGFR-β, which also form homo- and heterodimers [44,45,46]. PDGFRs are membrane-bound proteins consisting of five extracellular immunoglobulin-like domains, a transmembrane domain, a juxta-membrane domain, a kinase insert domain, and an intracellular kinase domain [46]. The PDGFR-αα is activated by all PDGF ligands, except PDGF-DD, PDGFR-ββ is activated by PDGF-BB and PDGF-DD, and PDGFR-αβ is activated by all PDGF ligands, except PDGF-AA. The activated receptors further initiate a complex Ras/MAP-kinase pathway [44].

PDGF has been originally isolated from platelets and considered as the major mitogen for mesenchymal cells, including fibroblasts, glial cells, and smooth muscle cells. PDGFs generation is tightly regulated, as they are stored in platelets and are released upon degranulation, as a response to thrombin or other stimuli [46,47]. Moreover, it has been proved that PDGFs are involved in a much broader range of functions, including vascular development by promoting proliferation and survival of vascular mural cells, and are expressed at undetectable or very low levels by a variety of cells, including monocytes, macrophages, lymphocytes, mast cells, connective tissue cells, pericytes, endothelial cells, and tumor cells [45,46,47].

#### 2.2.4. Angiopoietins

Ang are a family of proteins, including Ang1, Ang2, Ang3, and Ang4, which share a common amino-terminal coiled-coil domain and a carboxy-terminal fibrinogen-like domain [48]. Ang are related to vasculogenesis and vascular repair, with Ang1 and Ang2 as the major isoforms involved in regulating vascular homeostasis [49].

The biological activity of Ang occurs after binding with the cell-surface receptor tyrosine kinase, Tie2, which is preferentially expressed by endothelial cells and certain myeloid cells. There is an additional orphan receptor, Tie1, which is poorly characterized and presumed to act as a modulator of the Tie2 activity [50]. Tie2 is a unique receptor, as it is tonically phosphorylated in the vasculature, by contrast to other vascular tyrosine kinase receptors, which are activated at the site of angiogenesis. Moreover, the phosphorylation of Tie2 is regulated by a combination of ligands and cell-surface proteins, including Ang1, Ang2, Tie1, and vascular endothelial protein tyrosine phosphatase [51].

Ang1 is expressed by peri-endothelial mural cells, such as smooth muscle cells, and pericytes, fibroblasts, and other non-vascular stromal and tumor cells, serving as the main Tie2 agonistic ligand. Ang1 has a crucial role in the regulation of vessel maturation stabilization during embryonic development, vessel remodeling, maintenance of the normal vasculature throughout life, by promoting endothelial-mural cells interactions, endothelial cells survival, and non-permeability. Ang2, produced by the VEGF-stimulated endothelium, hypoxia, and shear stress, exhibits opposing actions to Ang1, as it promotes blood vessel wall destabilization through competitive inhibition to Tie2 and integrin activation. Additionally, Ang2 stimulates pericyte detachment, permeability, vascular regression, and lymphangiogenesis [48,49,50].

#### 2.2.5. Hepatocyte Growth Factor

Originally discovered as a potent mitogen for adult rat hepatocytes, HGF is a pleiotropic cytokine that exhibits a variety of cellular effects upon tyrosine phosphorylation of its receptor, c-Met [52,53]. HGF is secreted as a glycoprotein precursor, activated upon proteolytic cleavage. The active molecule comprises an αβ heterodimer, with the α chain functioning as a receptor binding, and the β portion necessary for activating the receptor and translating the binding into a cellular response. The receptor c-Met consists of an extracellular domain, composed of semaphorin or Sema domain, PSI domain, PTI domain, and an intracellular domain, composed of the tyrosine kinase. The composite structure of the receptor is completed by a transmembrane helix [54]. The receptor is expressed by endothelial cells, smooth muscle cells, and bone marrow-derived endothelial progenitor cells [52]. The binding of the HGF and c-Met promotes different cellular signaling pathways, which is involved in the regulation of tissue homeostasis under physiological conditions. Additionally, tissue hypoxia activates c-Met transcription, consequently leading to amplified HGF signaling [55]. The effects exerted by HGF stimulation of vascular endothelial cells include proliferation, migration, invasion, branching morphogenesis, protease production, and capillary tubes organization [56]. Moreover, it is involved in the regulation of various biological processes, including inflammation, tissue repair, morphogenesis, angiogenesis, tumor propagation, immunomodulation of viral infections, and cardio-metabolic activities [53]. As it can induce angiogenesis in the absence of vascular inflammation and increased permeability and, therefore, it does not require any prior triggers to exert its action, HGF is the most angiogenic factor [57].

#### 2.2.6. Hypoxia-Inducible Factor

Tissue perfusion homeostatic regulation is dependent on the activity of HIF-1, consisting of the HIF-1α and HIF-1β subunits. The activity of HIF-1 involves the regulation of oxygen homeostasis by activating the transcription of genes encoding proteins that increase oxygen delivery through angiogenesis and vascular remodeling or decrease oxygen consumption, by switching from oxidative to glycolytic metabolism. Moreover, it mediates hypoxia and ischemia tissue responses by activating genes encoding proangiogenic factors, such as VEGF, Ang, PlGF, PDGF [58]. While these factors are normally expressed, the activity of HIF can simultaneously induce the expression of a spectrum of angiogenic factors as a response to hypoxia [59]. Specifically, studies have shown that HIF-1α stimulates the expression of all VEGF isoforms, which will subsequently enhance the expression of the other proangiogenic factors, namely PlGF and FGF. Thus, it is safe to assume that in normal oxygen levels within cells and tissues, HIF-1α is degraded, thus inhibiting the VEGF gene and protein expression and stopping the cascade of proangiogenic factors [60].

### 2.3. Angiogenesis Inhibitors

Similarly, there is also a set of chemical signals that inhibit angiogenesis by disrupting blood vessel formation or supporting the removal of existing vessels, i.e., at the end of an inflammatory response [25]. Examples of such molecules that facilitate the termination of neovascularization through anti-apoptosis gene suppression are angiostatin, endostatin, platelet factor 4 (PF4), thrombospondin-1 and 2, interleukin-12, tumstatin, osteopontin, anti-angiogenic metargidin peptide, and endoglin silencing [22,25,61].

#### 2.3.1. Angiostatin

Angiostatin has proved to inhibit endothelial cell proliferation and migration, tube formation, neutrophil activation and migration, monocyte and macrophage migration, leukocyte recruitment, MMPs expression in endothelial cells, and tumor cell invasion by blocking plasminogen binding to CD26. Furthermore, angiostatin induces endothelial cell apoptosis and the production of anti-angiogenic factors, such as thrombospondin-1, and it attenuates VEGF expression by binding to ATP synthase, angiomotin, integrins, and annexin II or by preventing the transition between G2 and M phases in the cellular cycle [62].

#### 2.3.2. Endostatin

Endostatin is a C-terminal type XVIII collagen fragment, cleaved by the proteolytic activity of MMP-7 [63]. It has a broad spectrum of anti-angiogenic activities on endothelial cells, and the most common mechanism involves the inhibition of MMPs, which facilitate the proteolytic degradation of the extracellular matrix in the angiogenic process. Specifically, MMP-2, MMP-9, and MMP-13 are the targets for the inhibitory activity of endostatin [64]. Additionally, by binding to the α5 and αv-integrins, it inhibits the migration of vascular endothelial cells as a result of the MAPK signal blocking. Moreover, endostatin can also bind directly to VEGFR2, thus inhibiting the VEGF-induced phosphorylation and consequently down-regulating the receptor. The anti-angiogenic properties of endostatin can be attributed to the repression of cell cycle genes, such as cyclin D1, and antiapoptotic genes, leading to apoptosis in proliferating endothelial cells [63,64].

#### 2.3.3. Platelet Factor 4

PF4 is a 7.8 kDa protein that comprises 70 amino acids in its structure and is released from α-granule platelets in the process of platelet activation. It is the most abundant chemokine of the CXC chemokine subfamily [65], which is part of the chemokine family of small proteins with major roles in inflammation [66]. PF4 is a strong angiogenesis inhibitor [67], exhibiting its angiostatic effects by binding to different growth factors involved in the process [68]. Specifically, through heparin-dependent and -independent mechanisms, PF4 binds to the receptors of FGF2 and VEGF-A_165_, leading to downstream effects on endothelial cell migration and proliferation. Additionally, PF4 binds to endothelial cells through integrins and to thrombomodulin through its chain, thus being found in the blood vessel wall after the removal of the endothelium and attachment of platelets to the denuded basement membrane [69].

#### 2.3.4. Thrombospondin-1

Thrombospondin-1 is one of the five identified paralogs of thrombospondin, termed as thrombospondin-1-5. It is a 450 kDa glycoprotein and a major constituent of α-granules [68,70]. Thrombospondin-1 is known for its inhibitory effect on the migration, proliferation, and survival of endothelial cells and the formation of capillary tubes. Additionally, it is the first naturally occurring anti-angiogenesis factor that acts by displacing VEGF from its complex with heparan sulfate and further binding to VEGF. Its effects are exerted through CD36, CD47, and integrins [71] However, several studies have shown that considerably high concentrations of thrombospondin-1 promote angiogenesis [68,72].

### 2.4. Tumor Angiogenesis

In tumors, the nutrients and oxygen supply and the efficient metabolites drainage are provided through a complex tumor microvasculature network [73,74]. Until the early 1970s, it was generally believed that the vasculature of tumors resulted from an inflammatory reaction to necrotic tumor cells [75]. However, with the work of Judah Folkman, considered the father of angiogenesis research, novel insights have been brought to the understanding of tumor biology [76,77]. Specifically, Folkman hypothesized that in the absence of angiogenesis, tumors are restricted to microscopic sizes and enter a dormancy state, while proposing the anti-angiogenesis term for preventing the recruitment of new capillary sprouts into growing tumors and the possibility of using an antibody to a tumor angiogenic factor as an anticancer drug [75,76].

Tumor angiogenesis is achieved through a series of sequential steps that further lead to cancer development [78]. The angiogenic process is mainly initiated by the tumor itself, as the malignancy grows to a specific size and the cells become hypoxic [79]. Hypoxia is characterized by an O_2_ tension level lower than 5–10 mmHg, and it represents the fundamental initiator of tumor angiogenesis [73,80]. Consequently, hypoxic cancer cells secrete angiogenic molecules, including growth factors, cytokines, bioactive lipids, or matrix-degrading enzymes, which bind to the receptors found on the vascular endothelial cells of adjacent blood vessels and initiate the formation of new vessels [79,81,82]. The most important proangiogenic factor is VEGF, which promotes angiogenesis within tumors through its highly mitogenic effects [79,83]. After the VEGF-A level reaches a maximum concentration level at the leading edge of the vascular sprout, it binds to VEGFR2 and induces the migration of endothelial tip cells. Once activated, VEGFRs activates a series of downstream pathways involved in cell proliferation and survival, cytoskeleton rearrangement, and vascular permeability. Additional signaling molecules are involved, namely delta ligand-like 4 for controlling the tip-cell phenotype and Ang2 for destabilizing endothelial cell junctions [73,81]. Furthermore, MMPs released by activated endothelial cells degrade the endothelial basement membrane and the extracellular matrix and trigger the secretion of angiogenic growth factors, such as FGF-2, PDGF, and VEGF. After the release of FGF-2, PDGF, and TGF, resident fibroblasts present higher proliferation activities due to phenotype changes, and stimulate angiogenesis and metastasis by producing MMPs and PDGF-C. Additionally, the tumor microenvironment promotes the tumor-associated type II macrophage differentiation, which will release high amounts of VEGF, PDGF, FGF-2, MMPs, TNF, and inducible nitric oxide synthase-released reactive oxygen species. The subsequent process of vessel formation within tumors is similar to the physiological angiogenesis previously described [81,84]. Other mechanisms involved in tumor angiogenesis include vessel co-option, through which cancer cells grow along host tissue capillaries, intussusceptive microvascular growth, involving the formation new blood vessels from connective tissue pillars within the lumen, glomeruloid angiogenesis, which starts from glomeruloid bodies composed of vascular aggregates, basement membrane, and pericytes, postnatal vasculogenesis, consisting in the transformation of bone marrow-derived endothelial progenitor cells into endothelial cells, and vasculogenic mimicry, through which cancer cells act as endothelial cells and produce vessel-like structures [85].

**Figure 1:**
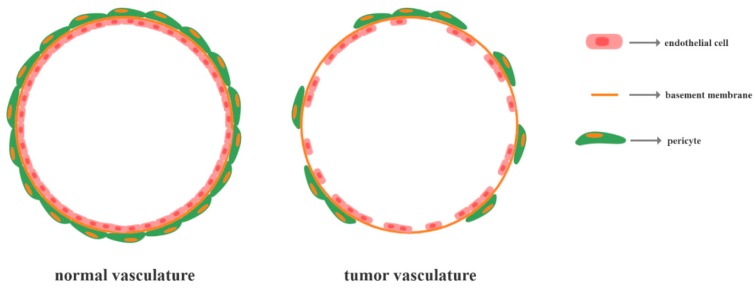
A schematic representation of the normal and tumor vasculature.

As tumor angiogenesis is characterized by an imbalance between pro- and anti-angiogenic modulators within the tumor microenvironment in the favor of the former [74], the vasculature of tumors is considerably different. By contrast to the normal vasculature, which is hierarchically distributed through a network of arterioles, capillaries, and venules, tumor blood vessels are highly disorganized, tortuous, and misshapen [86,87,88,89]. Furthermore, these vessels are mostly undifferentiated or immature, exhibiting reduced mural cell coverage by pericytes and an absent or collapsed lumen which terminates in open-ended blood-lakes [86,88,90]. Precisely, the endothelial cells of the vessels are not comprised of monolayers and they present disrupted junctions, forming abnormally loose associations with the existing pericytes and extending the cytoplasmic processes into the tumor tissue (Figure 1) [87,88]. Hence, tumor blood vessels are highly permeable which results in blood flow alterations and tumor cells diffusion into the interstitial space [88,89,91,92]. Consequently, these features increase the hypoxia in the tumor microenvironment, thus promoting the formation of novel vessels and the induction of metastasis [86].

## 3. Anti-Angiogenic Therapies

### 3.1. Conventional Anti-Angiogenic Drugs and Their Limitations

Based on the idea that the removal of the tumor vasculature through which nutrients and oxygen are supplied within the tumor could represent a potential anticancer therapy alternative, anti-angiogenic strategies have gained a great interest throughout the scientific world [93,94]. Specifically, anti-angiogenic therapies could avert the nutrition of tumor cells by eradicating the existing tumor blood vessels and hinder the formation of new ones, thus suppressing intravasation in primary tumors and prevent the angiogenic switch in metastases [95]. Moreover, such therapies could normalize tumor blood vessels to alleviate the hypoxia levels within the tumor microenvironment and, therefore, reduce the malignancy degree and increase the efficiency of conventional therapies [96,97,98]. In this context, anti-angiogenic agents or angiogenic inhibitors have emerged as a relatively new class of drugs that aim to disrupt tumor vascularization [99]. As VEGF-A is overexpressed in tumors and is associated with tumor progression, invasion, and metastasis, it represents the main target of anti-angiogenic drugs in cancer therapy, which are applied as VEGF-A and VEGFR2 inhibitors [100,101,102].

In the development of anti-angiogenic agents, there are four main strategies that are used: the inhibition of endogenous factors that promote the formation of blood vessels, the identification and application of natural angiogenesis inhibitors, the inhibition of molecules that promote the invasion of the surrounding tissue through tumor blood vessels, and the incapacitation of actively proliferating endothelial cells [25]. Moreover, such agents should target multiple angiogenesis triggers or pathways, disable the signal transduction pathways of vascular growth factors, exhibit a decreased tendency to induce resistance, stimulate anti-angiogenesis pathways, demonstrate specificity toward tumor angiogenesis, and have limited systemic toxicity and side effects [103].

Among the currently available anti-angiogenic drugs, bevacizumab, sunitinib, pazopanib, endostar, regorafenib, axitinib, sorafenib, ranibizumab, and aflibercept are the most applied in the treatment of various cancer types (Table 2) [100,102]. Additionally, several strategies rely on the administration of mTOR (the mammalian or mechanistic target of rapamycin) inhibitors, as mTOR is a protein kinase with fundamental roles in cell growth and proliferation [104]. Bevacizumab is a humanized monoclonal antibody that targets all VEGF-A isoforms to prevent angiogenic processes within tumors. It is the first anti-angiogenic drug approved for clinical application, and it has proved increased efficiency in multiple malignancy types, including colorectal cancer or glioblastoma [99,105,106,107,108]. The intravenous administration of bevacizumab results in increased VEGF blood levels, which is mostly antibody-bound and inactive [106]. However, treatment with bevacizumab has been associated with several side effects, including hypertension, proteinuria, and gastrointestinal perforations and bleeding. Additionally, post-treatment results have proved relapses due to increased invasion and resistance, thus requiring combined therapies for enhanced outcomes [107]. Sunitinib is a small molecule multiple tyrosine kinase inhibitor that targets VEGFR, PDGFR, stem cell factor receptor, colony-stimulating factor 1 receptor, fms-like tyrosine kinase receptor, and neurotrophic factor receptor [14,109,110,111,112]. The parallel inhibition of these receptors reduces tumor vascularization, resulting in the apoptosis of cancer cells [109]. Approved by the FDA in 2006, it has been used in the treatment of renal cell carcinoma and gastrointestinal stromal tumors [109,110]. The associated side effects of sunitinib are related to pulmonary toxicity, causing dyspnea and cough [110]. Pazopanib is a second-generation, small molecule multiple tyrosine kinase inhibitor that targets VEGF, PDGF, FGFR, stem cell factor receptor, colony-stimulating factor 1 receptor, and lymphocyte-specific protein tyrosine kinase receptor [14,111,113,114,115]. Pazopanib has been used in the treatment of renal cancer, epithelial ovarian cancer, and soft tissue sarcoma, but its administration has been associated with potential cardiovascular toxicities, namely high blood pressure and abnormal ventricular repolarization [115]. Endostar is the novel human recombinant version of endostatin (rh-endostatin) expressed in Escherichia coli. It was approved by the FDA in 2005 for the treatment of non-small cell lung cancer. Compared to endostatin, endostar exhibits considerably enhanced stability and biological activity [116,117].

Although these clinically approved anti-angiogenic drugs have proved a certain effectiveness towards reducing tumor angiogenesis by normalizing the hyperpermeable tumor vessels, metastasis and mortality continuously occur after treatment. As a consequence, inhibition of VEGFR or tyrosine kinase receptors are insufficient for stopping the neovascularization process [118,119]. Additionally, the administration of anti-angiogenic agents results in the development of an innate or adaptive resistance towards treatment partly due to changes in the immune microenvironment of the tumor [120]. As it is believed that the antiangiogenic drug resistance process is not caused by genetics, it has been suggested to be reversible and transient [121] The main mechanisms of anti-angiogenic drug-related resistance comprise revascularization, tumor vasculature protection, accentuated invasiveness of tumor cells, and increased metastatic manner through different modes of vascularization [122]. The processes underlying the antiangiogenic drug resistance of tumor cells comprise amplification of the pro-angiogenic genes, secretion of multiple proangiogenic factors, and recruitment of proangiogenic bone marrow–derived cells [123]. Therefore, novel anti-angiogenic strategies that could overcome the side effects and the resistance of these agents and with enhanced efficiency by targeting multiple cancer-related angiogenic mechanisms must be developed.

### 3.2. Novel Anti-Angiogenic Strategies for Cancer Treatment

Although the use of anti-angiogenic drugs has proved to effectively inhibit tumor progression, it cannot eradicate the tumor as a stand-alone strategy. In this regard, the combination of anti-angiogenesis agents and chemotherapy or immunotherapy could provide an effective cancer treatment [124].

While chemotherapeutic agents aim to kill cancer cells, they also affect normal host cells [125,126]. By associating them with anti-angiogenic drugs, the treatment outcome is considerably improved and the side effects are reduced [127,128]. Clinical studies have proved that the combination of bevacizumab and conventional chemotherapy leads to an increase in the survival and response rates in patients with gastrointestinal cancer, non-small cell lung cancer, breast cancer [129,130,131], and ovarian cancer [128]. Specifically, by implementing bevacizumab in the first-line treatment with gemcitabine and carboplatin for the treatment of recurrent ovarian cancer (OCEANS trial), the progression-free survival was improved, the maximum duration of follow-up being almost double [132,133]. Similar results have been obtained through the combination of bevacizumab with PEGylated liposomal doxorubicin, weekly paclitaxel, or topotecan for the therapy of platinum-resistant ovarian cancer. Specifically, the median progression-free survival was 6.7 months for bevacizumab-containing therapy, as opposed to 3.4 months for chemotherapy alone [132,134]. Furthermore, the efficacy of gemcitabine and cisplatin chemotherapy combined with the anti-angiogenic drug endostar was tested in advanced thymoma and thymic carcinoma, showing improved overall response rates, i.e., 75% vs. 42.9%, but not prolonged progression-free survival and overall survival when compared to chemotherapy alone [135]. The effects of endostar were also studied in metronomic chemotherapy using vinorelbine. Metronomic chemotherapy comprises the frequent administration of drugs at lower doses to inhibit neovascularization and induce tumor dormancy. The study was performed on a xenograft model of human lung cancer and it proved the efficiency of this strategy for anti-tumoral purposes, as it led to enhanced anti-tumor and anti-angiogenic effects without causing toxicity compared with either agent alone [136]. Apatinib is another anti-angiogenic agent that was assessed for its efficacy and safety in combination with an oral etoposide for the treatment of platinum-resistant or platinum-refractory ovarian cancer. This therapy showed promising efficacy with manageable toxicities, thus proving its potential for the treatment of ovarian cancer. In this manner, further studies in phase 3 trials are warranted [137]. Another clinical study focused on the combination of doxorubicin and apatinib or endostar as a strategy to treat sarcoma. Results showed significantly increased response rates, but no considerably different progression-free survival and overall survival than chemotherapy alone. Specifically, the overall response rate increased from 8.3% for chemotherapy alone to 17.3% by combining with endostar and 38.5% by combining with apatinib, while the disease control rate increased from 16.6% to 47.7% and 84.7%, respectively [138]. Nonetheless, it is essential that the chemotherapy coincides with the transient time window for vessel normalization, as prolonged or excessive administration of anti-angiogenic drugs reduces the microvascular density within tumors, thus compromising the delivery of the chemotherapeutic agent. In this manner, it is recommended that the anti-angiogenic agent be administered in intermittent cycles for inducing the re-normalization process [128].

Proangiogenic molecules have been associated with various immunosuppressive effects, including antigen presentation, T cell priming, T cell trafficking, and T cell tumor infiltration. Hence, the administration of anti-angiogenic drugs that could target these molecules would stimulate an immune response that can be exploited for antitumor immune responses. Specifically, the interactions of immune cells with tumors can be affected by these agents through the direct binding to their cognate receptors expressed by immune cells, induction of changes in protein expression on endothelial cells, promotion of vascular normalization, or reduction of neoangiogenesis [139]. Such observations have led to the development of anti-cancer therapies that combine anti-angiogenic agents and immunotherapy [90,140]. The inhibition of immune checkpoint molecules has become a clinically validated treatment for multiple tumors [141]. The immune checkpoint molecules, namely the programmed cell death protein 1 (PD-1) and its ligand the programmed death-ligand 1 (PD-L1) and cytotoxic T-lymphocyte antigen-4 (CTLA-4), are important components of the immune system which downregulate the T-cell immune function. By administering antibodies against these molecules, T cells are restored from their exhausted status and the tumor-killing activity is reestablished [141,142]. In this context, a preclinical study proved the synergetic effects of the combined anti-angiogenic and immune therapy. Specifically, the anti-PD-1 and anti-PD-L1 therapy sensitized and prolonged the efficiency of the anti-angiogenic therapy, while the anti-angiogenic agents improved the treatment by promoting the endothelial venule formation and vessel normalization to enhance the infiltration of cytotoxic T cells for tumor destruction [141,143]. Additionally, another study resulted in the alteration of tumor progression through the blockade of the immune checkpoint molecules which activated the CD4+ T-lymphocyte. These results were indicated through an increase in vessel normalization through the increased pericyte coverage, enhanced tumor vessel perfusion, and reduced vascular permeability [141,144]. Moreover, the anti-tumor potential of apatinib and anti-PD-L1 monoclonal antibody was tested on a murine lung cancer model using Lewis lung cancer cells. The combined treatment significantly inhibited tumor growth by continuously eliminating Foxp3+ regulatory T cells and myeloid-derived suppressive cells, dramatically decreasing the infiltration of tumor-infiltrating lymphocytes, and reducing the expression of PD-1 and PD-L1 [145].

While preclinical studies regarding anti-angiogenic therapies showed promising anti-cancer effects, the effects in patients are still limited. This discrepancy might be caused by the inability to recreate all the mechanisms involved in the complex processes of angiogenesis and to provide reliable tumor angiogenesis models that mimic the natural processes.

**Figure 2:**
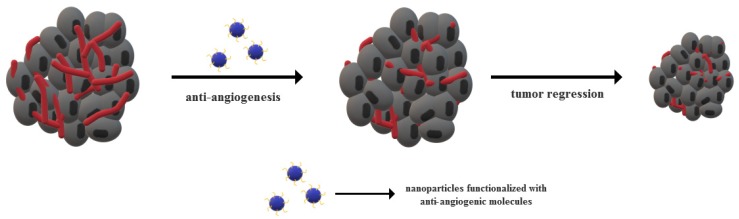
Nanotechnology and anti-angiogenesis combined strategies for cancer treatment.

The recent years have witnessed tremendous advancements of nanotechnology-based theranostic strategies for cancer treatment owing to the unique properties of nanomaterials in terms of size, surface area and energy, and physicochemical properties [146,147,148,149]. Significant progress is made in the scientific community to replace conventional cancer therapies with nanomedicine, and many studies provide evidence of the potential of nanomaterials to target tumor vasculature and inhibit angiogenesis [150]. Nanomedicine-based approaches mainly comprise the use of nanomaterials either with specific anti-angiogenic properties or as carriers for the delivery of anti-angiogenic and/or anti-cancer agents (Figure 2) [151,152]. As nanoparticles exhibit endothelial cells targeting abilities, they can efficiently deliver anti-angiogenic agents to the tumor site. Moreover, the use of nanoparticles further facilitates drug penetration into the tumor microenvironment and increase therapeutic efficacy by reducing the systemic toxicity [153,154]. The mechanisms of nanoparticle incorporation within the tumor mainly include ligand-mediated active targeting and passive targeting through the enhanced permeability and retention effects [151]. However, as only 0.7% of the administered nanoparticles are likely to reach the targeted site, novel strategies that are based on tumor vascular normalization must be developed. In this manner, the tumor blood flow would be enhanced, thus improving the perfusion and oxygenation of tumor cells and allowing for anti-cancer drug retention [151,154]. Examples of the intensively studied nanoparticles employed for the delivery of anti-angiogenic agents include cerium oxide, gold, silver, copper, silicate and silica-based, carbon-based, chitosan, and peptide-conjugated nanoparticles [153]. As it has been proven that nanoparticles properties, such as size and shape, directly influence angiogenesis processes within tumors, the fabrication process is essential for the outcome of the treatment [155].

## 4. Conclusions and Future Perspectives

Angiogenesis is the process that occurs mostly during embryonic and fetal development, but it can also happen in the adults in the event of wound healing, tissue growth, and the female reproductive cycle. Angiogenesis is the process of blood vessel formation from pre-existing ones, and there are three main mechanisms, namely sprouting, intussusception, and looping. The mechanism of angiogenesis is tightly regulated by highly specific angiogenic stimulators and inhibitors, as imbalances in the angiogenic process could lead to serious pathological conditions. The most common proangiogenic factors include VEGF, FGF, PDGF, Ang, HGF, and HIF, while anti-angiogenic factors include angiostatin and endostatin. As angiogenesis is an essential process for tumor growth, identifying proangiogenic molecules and blocking their activity through various approaches represents a potential anti-cancer therapy. However, as the administration of anti-angiogenic drugs does not eradicate the tumors, novel strategies are necessary to efficiently treat cancer. In this manner, combined strategies based on anti-angiogenesis and chemotherapy or immunotherapy have proved to be effective therapies that stopped tumor progression and metastasis. Moreover, nanotechnology has also been widely applied for the inhibition of angiogenesis within tumors. However, more studies are necessary in order to assess the potential applicability of nanomaterials-based strategies for clinical studies. Considering the heterogeneity of the tumor vasculature [156,157], one promising direction of nanotechnology-based anti-angiogenic strategy for cancer treatment is represented by the specific targeting of small arteries and veins within the tumor microenvironment for inducing necrosis.

## Figures and Tables

**Table 1:** The main angiogenic triggers involved in angiogenesis.

Protein Family	Component Proteins	Receptors
VEGF	VEGF-A VEGF-B VEGF-C VEGF-D VEGF-E PlGF	VEGFR1 (FLT1) VEGFR2 (KDR, FLK1) VEGFR3 (FLT4)
FGF	FGF-1 (acidic FGF) FGF-2 (basic FGF)	FGFR1 FGFR2 FGFR3 integrins heparin sulphate proteoglycans
PDGF	PDGF-A PDGF-B PDGF-C PDGF-D	PDGFR-α PDGFR-β
Ang	Ang1 Ang2 Ang3 Ang4	Tie2 Tie1
HGF	-	c-Met
HIF	HIF-1α HIF-1β	Aryl hydrocarbon receptor nuclear translocator
IGF	IGF-1 IGF-2	IGF1R IGF2R
TGFβ	TGF-*β*1 TGF-*β*2 TGF-*β*3	TGFBR1 TGFBR2
MMP	MMP1-23	-
TNF	TNF1-18	TNFR1 TNFR2

**Table 2:** The most common anti-angiogenic drugs applied for cancer treatment.

Anti-Angiogenic Drug	Targets	Uses	Side Effects
bevacizumab	VEGF-A isoforms	colorectal cancer glioblastoma	hypertension proteinuria gastrointestinal perforations gastrointestinal bleeding
sunitinib	VEGFR PDGFR stem cell factor receptor colony-stimulating factor 1 receptor fms-like tyrosine kinase receptor neurotrophic factor receptor	renal cell carcinoma gastrointestinal stromal tumors	diarrhea hypertension skin discoloration mucositis fatigue hypothyroidism
pazopanib	VEGF PDGF FGFR stem cell factor receptor colony-stimulating factor 1 receptor lymphocyte-specific protein tyrosine kinase receptor	renal cancer epithelial ovarian cancer soft tissue sarcoma	high blood pressure abnormal ventricular repolarization
